# Postoperative Outcomes Following Surgical Management of Secondary Peritonitis in a Referral Hospital in Eastern Venezuela

**DOI:** 10.7759/cureus.68130

**Published:** 2024-08-29

**Authors:** Ruth González-Guaimare, Yeisson Rivero, Adriana Hernandez-Velasquez, Enrique Avila-Liendo, Miguel Rivas-Perez, Cesar Estrella-Gaibor, Jackner Antigua-Herrera, Gabriel Gonzalez-Quinde, Debbye Machado-Paled, Silvia Agudelo-Mendoza, Tamara Rodriguez-Rugel, Wilson Garcia-Cazorla

**Affiliations:** 1 Department of Surgery, Universidad de Oriente, Núcleo Anzoátegui, Barcelona, VEN; 2 Department of Surgery, Universidad de Oriente, Núcleo Bolivar, Ciudad Bolivar, VEN; 3 Department of Surgery, Ministerio de Salud Pública, Hospital Esmeraldas sur Delfina Torres de Concha, Quito, ECU; 4 Department of Surgery, Instituto Tecnológico de Santo Domingo, Santo Domingo, DOM; 5 Department of Surgery, Hospital Leon Becerra Camacho, Milagro, ECU; 6 Department of Surgery, Universidad Catolica de Honduras, Tegucigalpa, HND; 7 Department of Surgery, Universidad Del Rosario, Bogota, COL; 8 Department of Surgery, Universidad Católica de Santiago de Guayaquil, Guayaquil, ECU; 9 Department of Surgery, Universidad de Cuenca, Cuenca, ECU

**Keywords:** appendicitis, laparotomy, mortality, morbidity, surgical management, secondary peritonitis

## Abstract

Background

Secondary peritonitis (SP) arises from direct contamination of the peritoneum by spillage from the gastrointestinal or urogenital tracts.

Objective

This research aimed to evaluate the clinical and epidemiological characteristics of patients with SP undergoing surgical management and to study potential factors associated with morbidity and mortality in a reference hospital in Eastern Venezuela.

Methodology

A retrospective cross-sectional study was conducted on patients aged 18 to 80 undergoing surgical treatment for SP at “Dr. Luis Razetti” University Hospital in Barcelona, Anzoátegui state, Venezuela, between January and December 2022. We calculated odds ratios to assess mortality risks based on the presence of postoperative complications.

Results

Analysis of 168 adult patients revealed a predominantly male population (n=110, 65.5%) with a mean age of 35.63 years (SD=14.34). Generalized peritonitis was observed in 126 cases (75%), primarily originating from the appendix (n=117, 69.6%). Postoperative complications occurred in 18 patients (10.7%); sepsis represented the most common associated complication (n=10, 43.5%). Patients with secondary peritonitis associated with acute appendicitis had a lower mortality rate (p=0.042). Additionally, laparotomy was associated with higher frequencies of complications (p=0.001) and mortality (p=0.025), while open appendectomy showed lower frequencies of complications (p=0.002) and mortality (p=0.035). Notably, patients experiencing postoperative complications had a significantly elevated risk of mortality (OR=98, 95% confidence interval = 21.74 - 441.69).

Conclusion

The most common source of SP was appendicular. Patients undergoing exploratory laparotomy for the management of SP had a higher frequency of complications and mortality, whereas those undergoing open appendectomy had lower rates of complications and mortality.

## Introduction

Inflammation of the peritoneum can be divided into primary, secondary, and tertiary peritonitis. Primary peritonitis results from bacterial translocation, hematogenous spread, or the iatrogenic contamination of the abdomen without a macroscopic defect in the gastrointestinal tract. By contrast, secondary peritonitis (SP) results from the direct contamination of the peritoneum by spillage from the gastrointestinal or urogenital tract [1]. Tertiary peritonitis is defined as a recurrent intra-abdominal infection that occurs 48 h after a well-succeeded control of a secondary peritonitis [2].

The frequency of SP compared with the other types has been poorly described. In a population of patients with carbapenem-resistant enterobacteria in an intensive care unit. About 25% of cases of peritonitis are attributable to the SP group. Among the sites of origin of peritonitis in order of frequency, we find the colon, appendix, stomach/duodenum, small intestine, and biliary tract [3].

Secondary peritonitis affects all populations regardless of age, gender, or geographic distribution, accounts for around 1% of all hospital visits, and is the second leading cause of sepsis worldwide. Mortality rates have been reported as high as 20%, particularly if not appropriately managed [4]. In some studies, the death rate exceeds that of respiratory infections, especially in the case of multidrug-resistant pathogens. In particular, postsurgical SP, being caused by nosocomial pathogens, is the most frequent cause of septic shock and multiple organ system failure in already compromised patients. These are, therefore, postsurgical complications with extremely poor prognosis and difficult therapeutic management if caused by multidrug-resistant pathogens. Therefore, its accurate diagnosis and prompt management are challenging, and surgery remains the gold standard for care [3].

The cornerstone of the treatment of SP is prompt elimination of the infectious focus, supported by intensive resuscitation and antimicrobial therapy. Prompt source control can be achieved by resection or restoration of the infectious or perforated visceral organ depending on the etiology and localization, the extent of the peritoneal contamination, and pre-existing comorbidities of the patient [5].

In Venezuela, there are no official reports of the incidence of SP, and studies on the topic are scarce, with the majority being over 10 years old. Some of these studies have reported high mortality rates of up to 22% [6]. Therefore, managing the disease in general surgery services presents a challenge to the work of the General Surgeon. Hence, the objective of this research was to evaluate the clinical and epidemiological characteristics of patients with SP undergoing surgical management and to study the possible factors associated with morbidity and mortality in these patients in a reference hospital in Eastern Venezuela.

## Materials and methods

A retrospective cross-sectional study was conducted to assess the clinical epidemiological characteristics of adult patients undergoing surgical treatment for SP at “Dr. Luis Razetti” University Hospital in Barcelona, Anzoátegui state, Venezuela, between January and December 2022.

The study enrolled patients aged between 18 and 80 years, admitted to the general surgery department of the aforementioned center, and surgically treated for SP. Patients older than 80 years were excluded to avoid potential confounders related to the susceptibility of this population. Pregnant patients and those diagnosed with sepsis upon admission were also excluded. Non-probability convenience sampling was utilized based on patients meeting these criteria.

The following data were collected from the medical records and epidemiological records of the included patients: sex, age, type of SP (localized vs. generalized), causes of SP, laboratory parameters including leukocytes, percentage of neutrophils, C-reactive protein (CRP), hemoglobin, platelets, surgical approach, frequency of complications, types of complications, and mortality. Between the complications, sepsis was based on the third international consensus definition for sepsis and septic shock [7].

The classification of anemia in patients was determined based on hemoglobin values according to the World Health Organization (WHO), considering anemia when the hemoglobin concentration is less than 12 g/dl in women and less than 13 g/dl in men [8,9]. Leukocytosis was defined as a leukocyte count greater than 11,000 white blood cells per microliter and was expressed as a percentage of patients with leukocytosis and leukopenia less than 4,000; neutrophilia was considered when the percentage of neutrophils was greater than 70% of the total leukocytes [10,11]. Thrombocytosis was considered when platelet values were more than 400,000 and thrombocytopenia when values were less than 150,000 [10,11]. The results related to the values of paraclinical studies were expressed as a function of the total available studies. Some patients did not have all the mentioned laboratory tests. Information corresponding to these variables was extracted from medical records and entered into a Microsoft Excel datasheet.

Data were presented as mean and standard deviation (SD) for quantitative variables and as percentages for qualitative variables. The association between qualitative variables was assessed using the chi-square test and the exact Mid-P test, and the association between quantitative variables was assessed with the Student t-test. The odds ratio (OR) was used to evaluate the risk of mortality based on the presence of postoperative complications using a 95% confidence interval (CI). A p-value <0.05 was considered statistically significant. For statistical analysis and reporting of results, IBM SPSS Statistics for Windows, Version 29 (Released 2023; IBM Corp., Armonk, New York, United States) and the OpenEpi version 3.01 online tool were utilized.

The present study was conducted following the norms and guidelines outlined in the STrengthening the Reporting of OBservational studies in Epidemiology (STROBE) statement and was approved by the general surgery department and the bioethics committee of the “Dr. Luis Razetti” University Hospital under Memorandum N° 254-HULR-2021.

## Results

**Figure 1:**
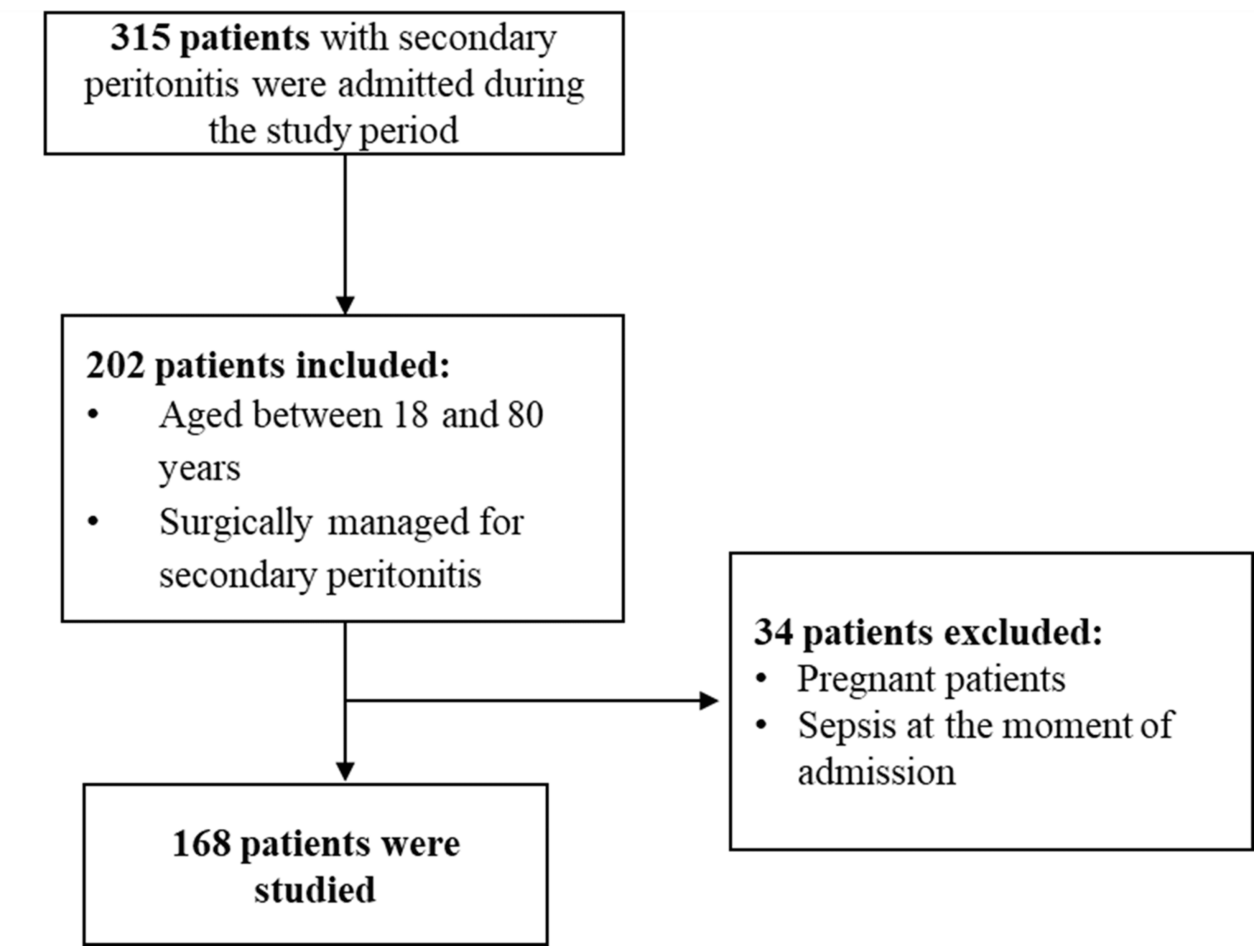
Patient selection flowchart

During the study period, 315 patients with a diagnosis of SP were admitted, of whom, after applying the inclusion and exclusion criteria, the results of 168 adult patients were analyzed (Figure 1). The majority were male, with 110 (65.5%) patients, and a mean age of 35.63 years and SD of 14.34, with 162 (96.4%) of the patients being under 65 years old. There was no statistically significant relationship found between gender and mean age (p=0.499).

Of the total patients evaluated, 126 (75%) presented cases of generalized peritonitis, with the primary cause being appendicular origin recorded in 117 (69.6%) patients, followed by perforation of the small bowel (n=38, 22.6%), postoperative peritonitis (n=6, 3.6%), and perforated gastrointestinal ulcer (n=7, 4.2%). The most common surgical approach was exploratory laparotomy in 77 (45.8%) patients.

Regarding paraclinical studies, 94 (56%) patients presented leukocytosis, 132 (78.6%) neutrophilia, and in 20 (17.9%) patients, the CRP test was reactive. Anemia was observed in 76 (45.2%) patients, being more common in male patients (75% vs 25%, p=0.006). Nineteen (11.3%) patients had thrombocytopenia, and 113 (67.3%) had thrombocytosis.

Table 1 presents the clinical-epidemiological characteristics of the studied patients and their relationship with the frequency of postoperative complications and mortality.

**Table 1:** Clinical-epidemiological characteristics of patients with SP CRP: C-reactive protein; SP: secondary peritonitis. a Continuous data are shown as the mean and standard deviation and categoric data as number (%) b Percentage values for laboratories were calculated based on the total available results. *Chi-square † Mid-P exact ‡ T-Student Values in bold are statistically significant

Characteristic	Postoperative complications			Mortality		
	Yes (N=18)	No (N=150)	p-values	Yes (N=15)	No (N=153)	p-values
Male gender	11(61.1)	99(66))	0.680*	9(60)	101(66)	0.640*
Age (mean ± SD)	38.5±13.5	35.29±14.4	0.372‡	37.8±15.5	35.4±14.2	0.542‡
Type of SP						
Generalized	13(72.2)	113(75.3)	0.773*	11(73,3)	115(75,2)	0.876*
Localized	5(27.8)	37(24.7		4(26,7)	38(24,8)	
Causes of SP						
Acute appendicitis	9(50,0)	108(72,0)	0.055*	7(46,7)	110(71.9)	0.042*
Perforated small bowel	7(38.9)	31(20.7)	0.081*	6(40)	32(20.9)	0.092*
Postoperative peritonitis	1(5.6)	5(3.3)	0.631†	1(6.7)	5(3.3)	0.498†
Perforated gastroduodenal ulcer	1(5.6)	6(4)	0.755†	1(6.7)	6(3.9)	0.612†
Paraclinical findings at admission						
Leukopenia	1(14.3)	17(13.6)	0.959	-	18(14.3)	-
Leukocytosis	6(85.7)	88(70.4)	0.384*	6(100)	88(69.8)	0.111*
Neutrophilia	7(87.5)	125(89.9)	0.825*	6(100)	126(89.4)	0.399*
Positive CRP	1(33.3)	19(17.4)	0.478†	3(30)	17(15.9)	0.12†
Anemia	4(57.1)	72(52.6)	0.813†	2(40)	74(53.2)	0.560†
Thrombocytopenia	1(5.6)	18(12)	0.415	-	19(12.4)	-
Thrombocytosis	5(27.8)	108(72)	<0.001*	3(20)	110(71.9)	<0.001†
Surgical approach						
Exploratory laparotomy	16(88.9)	72(48)	0.001*	12(80)	76(49.7)	0.025*
Open appendectomy	2(11.1)	75(50)	0.002†	3(20)	74(48.4)	0.035†
Laparoscopy	-	3(2)	-	-	3(2)	-

Postoperative complications were recorded in 10.7% (n=18) of the total patients studied; those complications were attributed to sepsis (n=9, 50%), intestinal obstruction (n=5, 27.8%), surgical site infection (n=2, 11.1%), bacteremia (n=1, 5.5%), and intra-abdominal abscess (n=1, 5.5%). Mortality was recorded in 15 (8.9%) patients.

It was observed that appendicitis was lower among patients who died compared with patients who survived (46.7% vs 71.9%, p=0.042). Furthermore, exploratory laparotomy as the surgical approach was more common in the group of patients with postoperative complications (88.9% vs 48%, p=0.001) and mortality (80% vs 49.7%, p=0.025), while open appendectomy was less common in the group of complications (11.1% vs 50%, p=0.002) and mortality (20% vs 48.4%, p=0.035). The details are presented in Table 1.

Patients who experienced postoperative complications had a significantly higher risk of mortality (OR=98, 95% CI = 21.74 - 441.69). Sepsis and surgical site infections were more common in patients with mortality with a statistically significant relationship. Details are presented in Table 2.

**Table 2:** Mortality based on the type of complications after SP SP: Secondary peritonitis. a Categoric data is shown as number (%) † Mid-P exact Values in bold are statistically significant

Type of complication	Yes (N=15)	No (N=153)	p-value
Sepsis	9 (60)	0	† <0.001
Intestinal obstruction	0	5 (3.3)	† 0.6228
Surgical Site infection	2 (13.3)	0	† 0.007
Intra-abdominal abscess	0	1 (0.7)	† 0.9107
Bacteremia	1 (6.7)	0	† 0.08929

## Discussion

The objective of this research was to evaluate the clinical and epidemiological characteristics of patients with SP undergoing surgical management and to study the possible factors associated with morbidity and mortality in those patients. It was found that the main cause of SP was of appendicular origin, primarily complicating with sepsis, and a significant relationship was found between patients undergoing laparotomy with a higher frequency of complications and mortality.

The average age found in our population (35 years) was slightly lower than that reported in other studies, where the average age ranged from 36 to 61 years; however, they coincide in that the majority were also male as in our population [12-14].

The main cause of SP recorded in our population was of appendicular origin, consistent with findings reported in the majority of studies on the subject, such as the study by Tagar et al. in 2023, where up to 46% of their patients with SP had appendicular causes [15], and Ramteke et al., also in 2023, where appendicular origin was the leading cause in 36% of cases [16]. However, Al Bisher et al. reported small bowel perforation as the primary source of SP (56%), which ranked second among our patients (22.6%) [13]. On the other hand, Novy et al. in 2023 reported that SP was mostly hospital-acquired, mainly postoperative peritonitis (50%), which ranked as the third cause in our patient series [17]. Additionally, Tagar et al. reported peptic ulcer perforation as the second leading cause (42%), while in our study, it ranked fourth with only 4.2% [15]. Similarly to other reports and as expected, the majority of cases corresponded to generalized SP, as reported by Pathak et al. [14].

Among the evaluated parameters, it was found that thrombocytosis was less frequent in patients with postoperative complications and those who died. Although this could have been an incidental finding as no relationship was found between thrombocytopenia and these outcomes, in patients with severe medical complications, thrombocytosis may be less frequent due to accelerated platelet consumption, bone marrow dysfunction, blood dilution, and effects of medical treatments [10,11]. Regarding the rest of the paraclinical parameters evaluated, no association was found between these and postoperative complications or mortality, similar to what was reported by Ramírez-Giraldo et al. in their series of 890 emergency laparotomies in 2023 [18].

Similar to our study, other series have reported exploratory laparotomy as the most common surgical approach in the management of SP [19]. This approach was also associated with a higher frequency of complications and mortality, which can be explained by the fact that these patients generally have a greater systemic inflammatory response, likely due to increased peritoneal contamination, exposure to greater tissue trauma, and longer duration of surgery [5].

Outcomes in patients with SP

In our study, 10.7% of patients experienced postoperative complications arising from SP, a proportion lower than that reported in other studies such as Mabewa et al., who reported complications in 36% of the 97 evaluated patients, among which surgical site infections (SSIs) and septicemia were the most common, similar to what was reported in our study [20]. Other studies have reported up to 40% of complications in patients with similar causes [19].

The main complication in our study resulting from SP was sepsis, which is not surprising as large-scale epidemiologic studies show that SP is the second leading cause of sepsis worldwide [21]. Tagar et al. reported surgical site infection as the main complication in 44%, while in our study, SSIs also rank among the top complications in the third place at 13% [15].

The mortality rate in patients with SP varies according to published studies, with rates ranging from 6.2% to 29.7% [14,16,17,22]. The mortality frequency in our study of 8.9% falls within the lower end of the range reported in the literature. The relationship between study variables and mortality frequency was evaluated, finding that patients whose cause of SP was appendicular had a lower frequency of deaths, similar to what was reported by Pathak et al., where in their study with 235 patients with SP, only those with appendicular origin did not report deaths [14]. Acute appendicitis is usually diagnosed and treated earlier compared to other causes of SP, such as intestinal perforations. Therefore, this patient may have a lower initial bacterial burden and less spread of infection. Additionally, appendectomy has lower risk of complications compared to surgeries that may be required for other causes of SP [23].

Other factors such as sex, diffuse presentation of peritonitis, and origin of SP, among others, were not associated with higher mortality, similar to what has been reported in previous studies [16]. Meanwhile, other factors such as age, which have been widely associated with mortality, including in SP, as presented by Arvaniti et al. [24], were not significantly associated with higher mortality rates in our study, similar to what was reported by Mabewa et al. [20].

In our study, patients with post-SP complications had a higher risk of mortality. In patients with SP, developing complications after surgery significantly increases the risk of death. This is because complications can exacerbate the existing infection and prolong recovery, making it more challenging for the body to combat the illness. Minimizing postoperative complications is essential for improving patient survival rates in these cases, which has been reported in other studies such as that of Pathak et al. [14].

Limitations and strengths

One significant limitation of the research study was that a considerable percentage of patients did not have all the required laboratory tests. For instance, only 66.7% had CRP values, which is one of the most valuable preoperative markers for identifying sepsis as a complication of SP [25], while others like procalcitonin were not recorded, partly due to the high cost of the test and limited access for many patients. Additionally, the low number of recorded cases of complications and mortality may have limited the detection of some significant relationships with the studied variables. Therefore, prospective cohort studies comparing patients with and without SP should be conducted, incorporating a broader range of variables to identify risk factors associated with this condition. These variables could include comorbidities, length of hospital stay, and specialty of management, as reported by other studies [12,13]. Additionally, these studies should include bacterial cultures to identify the main bacteria involved in SP and their sensitivity patterns [17]. This approach will provide a comprehensive understanding of the factors contributing to SP and inform more targeted interventions and treatment strategies. However, a strength of the study is that it examined a significant sample of patients, marking the first study on SP in the eastern region of the country. Through this study, local trends in the process of SP in our patients could be determined, and some factors associated with its morbidity and mortality could be identified.

## Conclusions

The clinical-epidemiological characteristics and causes of SP were similar to what has been reported in the literature. Mostly male patients were observed, with the primary cause of SP being appendicular in origin. Patients undergoing exploratory laparotomy for the management of SP had a higher frequency of complications and mortality, whereas those undergoing open appendectomy had lower rates of complications and mortality. More studies with prospective cohort designs are recommended to identify other risk factors associated with the morbidity and mortality of SP. These studies can help in designing prevention and management strategies.
